# Statistical inference for dependence networks in topological data analysis

**DOI:** 10.3389/frai.2023.1293504

**Published:** 2023-12-14

**Authors:** Anass B. El-Yaagoubi, Moo K. Chung, Hernando Ombao

**Affiliations:** ^1^Statistics Program, King Abdullah University of Science and Technology, Thuwal, Saudi Arabia; ^2^Department of Biostatistics and Medical Informatics, University of Wisconsin-Madison, Madison, WI, United States

**Keywords:** topological data analysis, time series analysis, simulating topological dependence patterns, spectral analysis, simulation-based inference

## Abstract

Topological data analysis (TDA) provide tools that are becoming increasingly popular for analyzing multivariate time series data. One key aspect in analyzing multivariate time series is dependence between components. One application is on brain signal analysis. In particular, various dependence patterns in brain networks may be linked to specific tasks and cognitive processes. These dependence patterns may be altered by various neurological and cognitive impairments such as Alzheimer's and Parkinson's diseases, as well as attention deficit hyperactivity disorder (ADHD). Because there is no ground-truth with known dependence patterns in real brain signals, testing new TDA methods on multivariate time series is still a challenge. Our goal here is to develop novel statistical inference procedures via simulations. Simulations are useful for generating some null distributions of a test statistic (for hypothesis testing), forming confidence regions, and for evaluating the performance of proposed TDA methods. To the best of our knowledge, there are no methods that simulate multivariate time series data with potentially complex user-specified connectivity patterns. In this paper we present a novel approach to simulate multivariate time series with specific number of cycles/holes in its dependence network. Furthermore, we also provide a procedure for generating higher dimensional topological features.

## 1 Introduction

Topological data analysis (TDA) has witnessed many important advances over the last twenty years that aim to unravel and provide insight to the “shape” of the data (Edelsbrunner et al., 2002; Edelsbrunner and Harer, 2008; Wasserman, 2018; Chazal and Michel, 2021). The development of TDA tools such as barcodes and persistence diagrams (Ghrist, 2008; Bubenik, 2015; Adams et al., 2017) have opened many new perspectives for analyzing various types of data (Umeda, 2017; Gholizadeh and Zadrozny, 2018; Motta, 2018; Xu et al., 2021; Leykam and Angelakis, 2023). These tools enable practitioners to grasp the topological characteristics inherent in high-dimensional data, which often remain beyond the reach of classical data analysis methods. However, a primary constraint of TDA tools is the absence of robust statistical inference techniques. Our goal in this paper is to introduce a simulation-based inference approach to address this limitation.

Many data sets exhibit a temporal structure (e.g., brain signals, economic data, climate data). In recent years, there has been a noticeable transition from primarily utilizing TDA techniques on clouds of to increasingly applying them on dependence networks of multivariate time series data, particularly for multivariate brain signals such as electroencephalograms (EEG) and local field potentials (LFP) (El-Yaagoubi et al., 2023). Rather than using TDA techniques on a cloud of points via a time delay embedding transformation, it is suggested that the multivariate time series be transformed to its dependence network where the nodes correspond to the time series components and the weight on the edges depend on the intensity of the statistical dependency between any given pair of time series in a network. There is currently no systematic method nor statistical model for conducting simulations (for the purpose of statistical inference or evaluating TDA methods) on networks with complex dependence structure. This is a serious limitation because simulations can form a basis for inference as well as evaluation of TDA methods for sensitivity, specificity, predictive ability. In this paper, we will develop an easily implementable method for simulating data with complex dependence patterns. Thus, the main contributions of the proposed simulation method are the following: (a) they enable a rigorous evaluation and comparison of data analytic methods and (b) provide tools for conducting proper statistical inference (in particular, hypothesis testing).

In the literature, the topology of the brain network (structural and functional) is believed to be organized according to principles that maximize the flow of information and minimize the energy expenditure for maintaining the entire network, such as small world networks (Sporns, 2013; Pessoa, 2014; Muldoon et al., 2016; Henry et al., 2020; Fathian et al., 2022). This topological structure of the brain network can be altered by various conditions such as attention deficit hyperactivity disorder (ADHD), Alzheimer's and Parkinson's diseases. Topological tools have been developed to assess and analyze the topological patterns of different groups' brain networks (e.g., healthy control vs. pathological), as well as quantifying the impact of these disorders on brain organization.

The goal of topological data analysis for time series is to provide computational tools that can assess the topological features present in the dependence network of a multivariate time series. Through the use of persistent homology theory, TDA provides a framework for analyzing the topological features, such as connected components, holes, cavities, etc. that are present in the network (Wasserman, 2018; Chazal and Michel, 2021).

**Figure 1:**
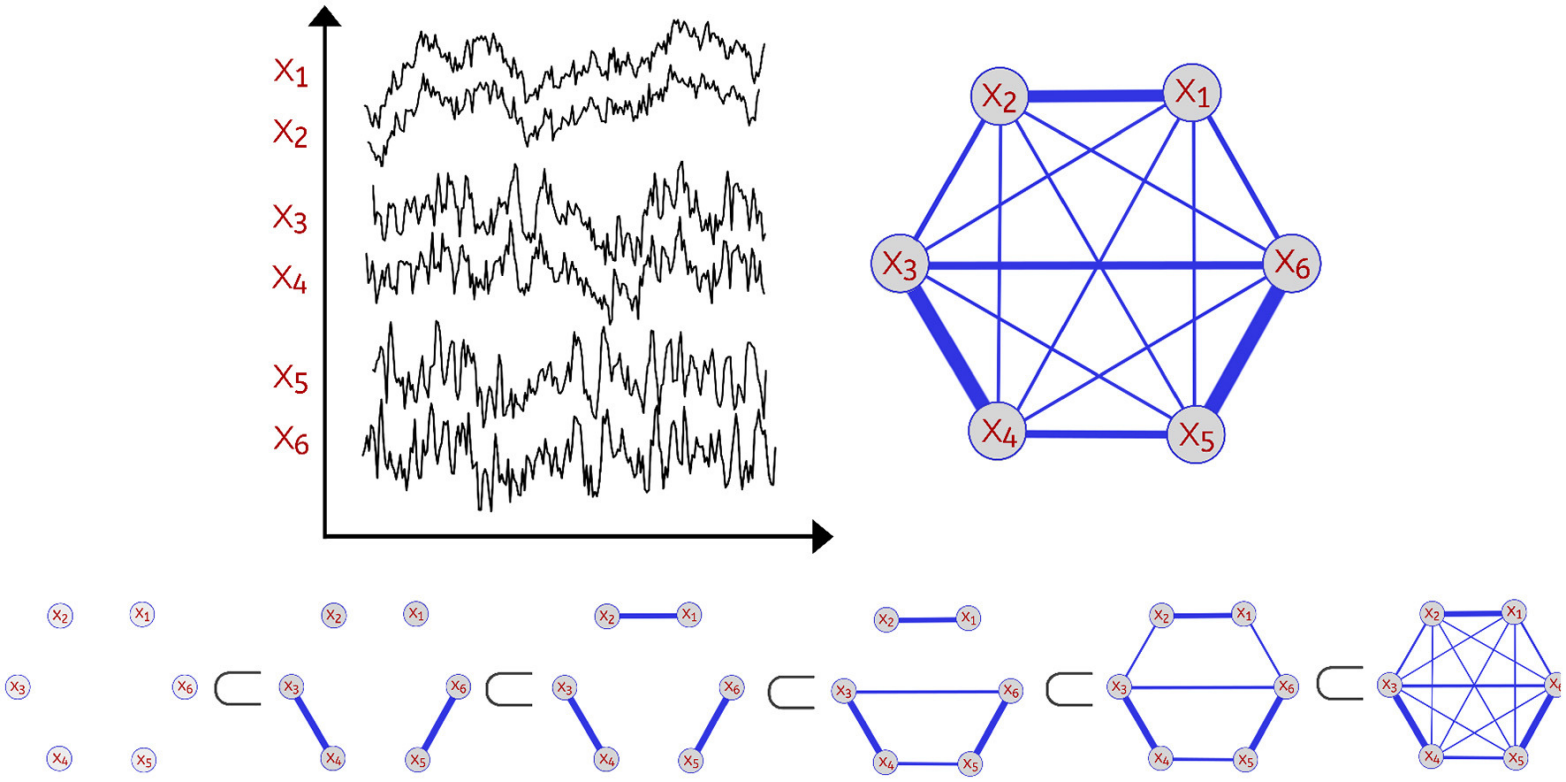
Example of a multivariate time series with P=6 channels **(Top Left)**. Dependence network of the multivariate time series **(Top Right)**. Considering this weighted network with *P* nodes (brain regions) with corresponding time series *X*_1_(*t*), …, *X*_*P*_(*t*), we define the distance between two time series components as a decreasing function of the strength of dependence. Thus, a pair of channels with weak dependence has a large value of the distance measure. For every threshold ϵ, a network with edges with weights not exceeding ϵ is constructed leading to an increasing sequence also known as Vietoris-Rips filtration **(Bottom)**. As the threshold ϵ grows, there's birth and death of various topological features.

In order to analyze the topological features present in the various dependence networks, we consider the homology of the filtration (increasing sequence of thresholded networks) obtained from these dependence networks, also known as Vietoris-Rips filtration. Let $X\left(t\right)={\left[X_{1}\left(t\right),\ldots ,X_{P}\left(t\right)\right]}^{T}$ be the observed brain signals at *P* different locations at time *t*∈{1, ...*T*}. One can define the dependence network to be a weighted graph with weights between nodes *p* and *q* to be some dependence measure between the observed time series components *X*_*p*_(*t*) and *X*_*q*_(*t*) as seen in Figure 1.

**Figure 2:**
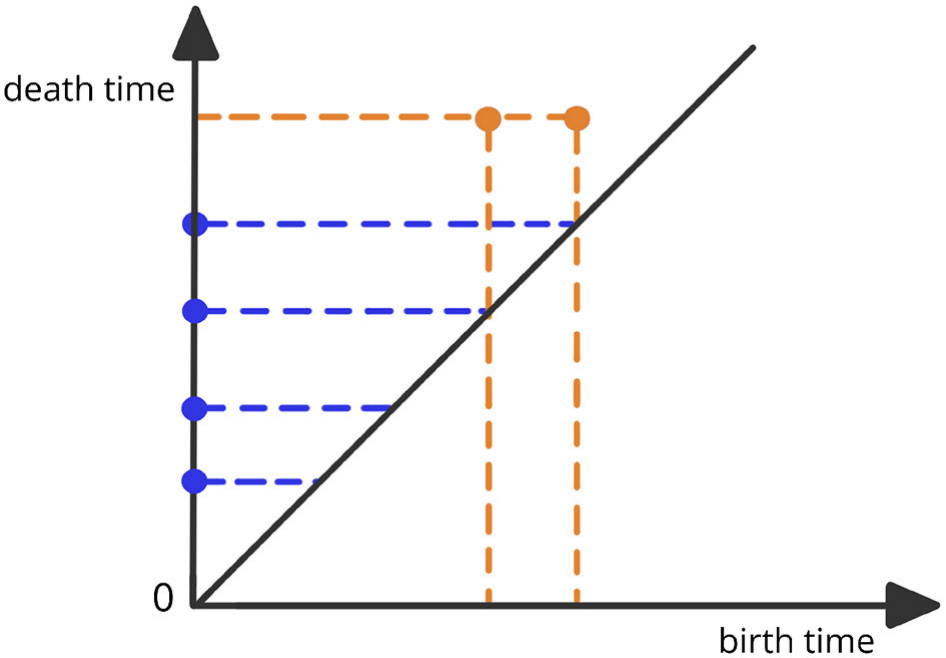
Example of a persistence diagram corresponding to the previous Vietoris-Rips filtration. The various dots correspond to different birth-death pairs (*b*_ℓ_, *d*_ℓ_) with birth time in the x-axis and death time in the y-axis.

Since the Vietoris-Rips filtration relies on the notion of distance, we then define distance between brain channels at locations *p* and *q* to be a decreasing function of the strength of a dependence metric between this pair of channels. For example, using coherence or correlation as dependence measures, one can use the transform *g*(*x*) = 1−|*x*| (where *x* is correlation or coherence). A comprehensive discussion on dependence in the spectral domain (e.g., coherence) is given in Ombao and Pinto (2022). Therefore, using this measure of distance, the Vietoris-Rips filtration (see Hausmann, 2016) is constructed by connecting nodes that have a distance less or equal to some given threshold ϵ, which results in the following filtration:


(1) $$\begin{array}{l} X_{\epsilon_{1}}\subset X_{\epsilon_{2}}\subset \cdots \subset X_{\epsilon_{n}}, \end{array}$$

where Xϵ represents the simplicial complex at the threshold level ϵ. This complex is defined to be the combination of all *k*-simplices (nodes, edges, triangles etc.) of brain channels that are within a maximum distance of ϵ from each other. The thresholds for distance are defined as 0 <ϵ_1_ <ϵ_2_ <⋯ <ϵ_*n*−1_ <ϵ_*n*_. For a visual illustration, refer to Figure 1. The objective of this approach is to assess the scales at which topological features (connected components, cycles, holes, and so on) appear (birth time) and then vanish (death time) (Wasserman, 2018; Chazal and Michel, 2021). The Vietoris-Rips filtration can be a complex object. Therefore, the most common topological summary being utilized is the persistence diagram (PD) which is a diagram that represents the times of births and deaths of the topological features in the VR filtration (see Figure 2). Every birth-death pair is represented by a point in the diagram, e.g., (*b*_1_, *d*_1_), (*b*_2_, *d*_2_), …, where *b*_ℓ_ is the birth time of the ℓ-th feature and *d*_ℓ_ is the death time of the ℓ-th feature. The points in the PD are colored based on the dimension of the feature they correspond to (e.g., one color for the connected components, another color for the cycles etc.).

There are multiple approaches that are available for modeling and generating multivariate time series data, each offering distinct advantages and limitations. For instance:

- **Parametric VARMA models:** Parametric Vector Autoregressive Moving Average (VARMA) models exhibit flexibility by accommodating dependencies on lagged values of each variable and interactions between terms or variables. This versatility enables the representation of contemporaneous and lagged dependencies, providing a comprehensive modeling framework (Shumway and Stoffer, 2017, Gorrostieta et al., 2018).
- **Gaussian process-based models:** GP provide a flexible framework for capturing complex and non-linear relationships. These models accommodate non-stationary processes, allowing changes in mean and covariance structures over time. Moreover, they allow the incorporation of prior knowledge through the selection of covariance functions (kernels) (Mohammadi et al., 2019).
- **Copula-based models:** These model are effective in modeling tail dependence, particularly in extreme events where variables exhibit dependencies in the tails of their distributions. This capability is crucial for understanding rare and extreme events and enables the modeling of non-Gaussian marginal distributions, addressing real-world scenarios where individual time series do not follow Gaussian distributions (Brechmann and Czado, 2014).
- **Machine learning models:** These data-driven models, such as Generative Adversarial Networks (GANs) offer innovative and powerful methods for generating synthetic multivariate time series, showcasing high flexibility and versatility. They excel in learning complex patterns and dependencies, including non-linearities and temporal dynamics (Snow, 2020).

However, a notable limitation shared by these existing models is their inability to generate multivariate time series data with predetermined patterns in the dependence network, such as a specific number of cycles. Cycles represent situations where nearby channels exhibit correlations or dependencies, while channels further apart may not have direct connections but are linked through intermediary channels in a cyclic manner. The number of cycles can vary, ranging from none to multiple cycles. Driven by the challenges at hand, this paper introduces an innovative method for simulating multivariate time series data with predetermined dependence patterns. The dual purpose is to enable formal statistical tests on complex topological networks and to evaluate the effectiveness of Topological Data Analysis (TDA) methods in the realm of multivariate time series data. Table 1 provides a comprehensive overview, summarizing both the strengths and limitations of existing approaches alongside our proposed method.

**Table 1:** Comparison between our approach and existing approaches in multivariate time series modeling.

**Approach**	**Strengths and advantages**	**Limitations and challenges**
Parametric VARMA	•Captures lead-lag dependencies •Easily interpretable	•Assumes stationarity •Limited to linear dependencies
Gaussian processes	•Captures non-linear dependencies •Allows incorporation of prior knowledge	•Difficulty in imposing arbitrary dependence patterns •Limited scalability
Copula-based	•Models tail dependence •Captures non-linear dependencies •Accommodates a wide range of marginal distributions	•Difficulty in imposing arbitrary dependence patterns •Handling time dependence can be challenging
Machine learning	•Captures complex and non-linear dependencies •Captures lead-lag dependencies •No stationarity assumption	•Requires training data •Prone to overfitting •Limited interpretability
Our approach	•Captures any dependence pattern •Easily interpretable	•Requires definition of a dependence graph •Choice of weight decay function

In this paper, we introduce an innovative method that leverages mixtures of latent second-order autoregressive processes to generate multivariate time series data showcasing diverse connectivity patterns within the dependence network. In Section 2, we provide a concise overview of AR(2) processes, detailing the generation of various dependence patterns by mixing these AR(2) processes using carefully selected weights. We also demonstrate how the resulting persistence diagram effectively identifies such patterns. In Section 3, we explain how to generate multivariate time series data with more general patterns, and investigate the sensitivity of this approach at various signal-to-noise ratios. Finally, in Section 4, we use our approach to carry out simulation-based inference based on the notion of total persistence.

## 2 Modeling dependence patterns in multivariate time series

In neuroscience, the concept of the nervous system as a (structural and functional) network of interconnected neurons is now well established (Friston, 2011; Sporns, 2013; Nakagawa and Deco, 2015; Fan et al., 2016). Many brain investigations have led to countless discoveries concerning the brain's anatomical and functional organization. The ongoing scientific endeavor in neuroscience to map the complicated networks of the human brain with increasing accuracy has been primarily due to the technological advances in brain imaging techniques that have resulted in new statistical techniques that aim to study and analyze various patterns in these complex networks. Such methods not only help neuroscientists understand the segregation of brain functions but also the integration of information processing. As a result, the validity of such novel techniques must be evaluated in terms of various metrics such as false positive or false negative rates, type I and type II errors, power of the test (ability of the test to detect differences in patterns between groups when they truly exist). It is impossible to evaluate/assess such approaches without a proper method for generating multivariate time series data with ground truth patterns in its dependence network. This is readily accomplished via extensive computer simulations under various settings of the truth (user-specified ground truth for the dependence networks).

Given the intrinsic complexity of brain signals, which are considered to be a superposition of random oscillations at specific frequencies or frequency bands, it can be challenging to discover and analyze the interrelationships between distinct time series components. As a result, this paper will adopt a frequency-specific strategy to generate meaningful simulations. We will develop a method where the multivariate time series data with dependency connections that are allowed to vary across frequency bands [again, we refer the reader to Ombao and Pinto (2022) for a discussion on spectral metrics for dependence]. For this reason, we will consider coherence as our frequency-specific dependence measure, since it can capture specific oscillations that are common to components in a network of signals. The typical spectral approach for analyzing brain data is to first estimate the spectral matrix, then construct the connectivity network using a spectral dependence measure, usually, coherence or partial coherence (Bowyer, 2016; Hu et al., 2019).

In the following subsections, we will generate multivariate time series data with specific dependence patterns. While numerous methods exist for generating multivariate time series as mixtures of random oscillations, our primary focus will be on utilizing second-order autoregressive processes that are concentrated around a specific frequency band.

### 2.1 Autoregressive processes of order 2

Electrophysiological signals are modeled as mixtures of many random oscillations. Here, each random oscillation with a desired power spectrum will be modeled as a second-order autoregressive process [AR(2)]. One advantage of using AR(2) processes as building blocks for a time series is their ability to represent oscillations at precise frequency bands. See Prado et al. (2001) and Granados-Garcia et al. (2021).

A linear mixture of second order autoregressive processes [AR(2) processes] can be used to simulate the brain oscillatory activity at specific frequency bands. An AR(2) process with a spectral peak at pre-specified frequency and bandwidth can be used to describe a latent process as follows:


(2) $$\begin{array}{ll} Z\left(t\right) & =\phi_{1}Z\left(t-1\right)+\phi_{2}Z\left(t-2\right)+W\left(t\right) \end{array}$$

where *W*(*t*) is white noise process with *EW*(*t*) = 0 and *VW*(*t*) = σ^2^; the relationship between the AR(2) model parameters ϕ_1_ and ϕ_2_ and the spectral properties, namely the frequency peak and bandwidth, will be derived as follows. Note that Equation 2 can be rewritten as $W\left(t\right)=\left(1-\phi_{1}B^{1}-\phi_{2}B^{2}\right)Z\left(t\right)$ where the back backshift operator *B* is defined as *B*^*k*^*Z*(*t*) = *Z*(*t*−*k*) for *k* = 1, 2. The AR(2) characteristic polynomial function is:


(3) $$\begin{array}{l} \Phi \left(r\right)=1-\phi_{1}r^{1}-\phi_{2}r^{2}. \end{array}$$

**Figure 3:**
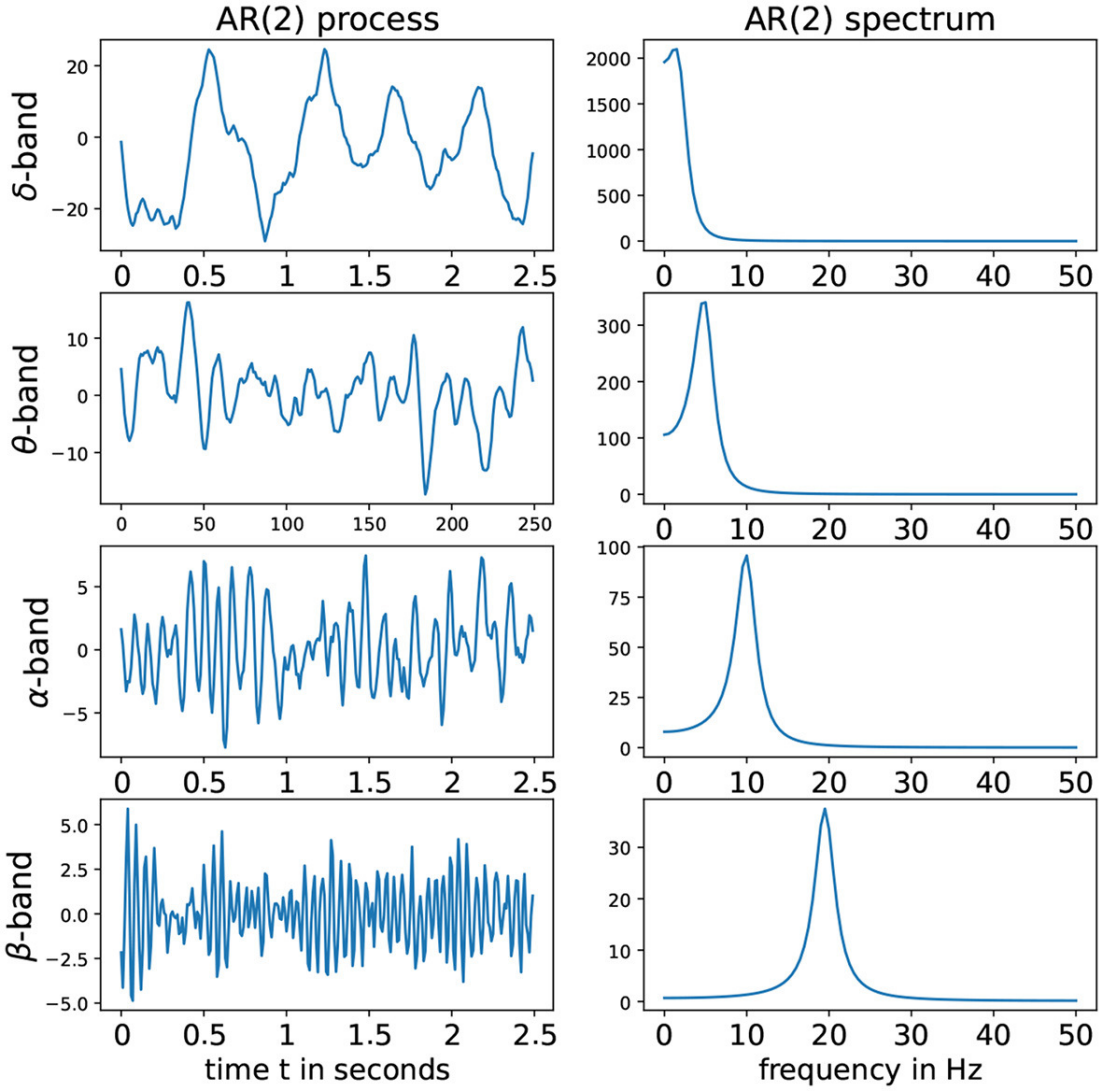
**(Left)** AR(2) processes for different frequency bands. **(Right)** Corresponding spectra. From top to bottom we have: delta-band with peak frequency at 2 Hz, theta-band with peak frequency at 5 Hz, alpha-band with peak frequency at 10 Hz, alpha-band with peak frequency at 2Hz and beta-band with peak frequency at 19.5 Hz with sampling rate (SR) of 100*Hz*.

Denote the roots of the Φ(*r*) to be *r*_1_ and *r*_2_. To fulfill the conditions of stationarity and casuality, the roots should lie outside of the unit circle on the complex plane, i.e., |*r*_1_|>1 and |*r*_2_|>1. Consider the case when *r*_1_ and *r*_2_ are (non-real) complex-valued. Hence these can be expressed as *r*_1_ = *M*exp(*i*2πψ) and *r*_2_ = *M*exp(−*i*2πψ) where the phase ψ∈(0, 0.5) and the magnitude *M*>1 to satisfy causality (Shumway and Stoffer, 2017). For this latent process *Z*(*t*), suppose that the sampling rate is denoted by *SR* and the peak frequency is *f*∈(*f*_min_;*f*_max_). Then the roots of the AR(2) latent process must have the phase ψ = *f*/*SR*. In practice, if the sampling rate *SR* is 100 Hz and we wish to simulate an alpha-band latent process where the peak is at 10 Hz, then it is necessary to set ψ = 10/100 and the root magnitude *M* to some number greater than 1 but “close” to 1 so that the spectrum of *Z*(*t*) is mostly concentrated on the frequency band *f*-Hz. The corresponding AR(2) coefficients are derived to be $\phi_{1}=\frac{2}{M}\cos\left(2\pi \psi \right)$ and $\phi_{2}=-\frac{1}{M^{2}}$. Some examples of such stationary AR(2) processes as well as their corresponding spectrum is given in Figure 3.

### 2.2 Mixtures of AR(2) processes

In TDA applications, Rips-Vietoris filtrations are often applied to multivariate time series data (Umeda, 2017; Gholizadeh and Zadrozny, 2018; El-Yaagoubi et al., 2023). These filtrations are often constructed from clouds of points, or from a weighted network. Traditionally, due to their stochastic nature brain signals have often been modeled using their underlying dependence networks. For instance in Bullmore and Sporns (2009), Henry et al. (2020), and Fathian et al. (2022), the authors use graph theoretical methods on complex brain networks.

To replicate a specific dependency pattern in the dependence network of a multivariate time series, it is necessary to emulate the decay in the dependence structure as time series components get farther away from each other. First sample a graph *G* = (*N, E*) with the desired structure (i.e., cycles or holes etc.), second define the observed time series components as mixtures of the latent processes, such that components near to each other in the graph share more latent processes, which makes them more dependent on one another, while components far away in the graph will share fewer latent components, resulting in lower interdependence. Let *Z*_*p*_(*t*) be the latent iid AR(2) processes centered around a specific frequency band. Therefore, to generate multivariate time series with a desired dependence patterns (as defined by the graph *G*) the following model is suggested:


(4) $$\begin{array}{ll} Y_{p}\left(t\right) & =\overset{P}{\underset{q=1}{\sum }}W_{p,q}Z_{q}\left(t\right)+\epsilon_{p}\left(t\right) \end{array}$$

(5) $$W_{p,q}\;=\{\begin{array}{ll} \frac{1}{1+d_{G}(p,q)}, & \text{ if }d_{G}(p,q)\leq K,\\ 0, & \text{ if }d_{G}(p,q)>K \end{array}$$

We generate a *P*-dimensional vector of observations $Y\left(t\right)={\left[Y_{1}\left(t\right),...,Y_{P}\left(t\right)\right]}^{T}\in {\mathbb{R}}^{P}$ that is a linear mixture of *P* latent iid AR(2) processes *Z*_1_(*t*), ..., *Z*_*P*_(*t*) according to Equation 4, whith *EZ*_*p*_(*t*) = 0, and *VZ*_*p*_(*t*) = 1. The mixing weights *W*_*p, q*_ contain the information about the importance of the *q*-th latent AR(2) process *Z*_*q*_(*t*) in the *p*-th observed component *Y*_*p*_(*t*), and as defined by Equation 5, the weights are chosen to be inversely proportional to the distance *d*_*G*_(*p, q*) between the nodes in the graph, and *K* being the maximum distance threshold that is considered, in practice we take *K* = 2 or *K* = 3. Theoretically, any distance-decreasing function might be used. However, selecting a faster decay (such as exponential decay) could result in a too-sharp decrease in the dependence based distance, making it more challenging to identify the topological features in the filtration.

It is essential to consider that the complexity of the dependence pattern directly impacts the required size of the sampled graph (denoted by *P*). This necessity arises from the need to attain higher resolution, ensuring that the sampled nodes adequately cover the entire manifold. Consequently, our approach may encounter limitations when generating intricate patterns within the dependence network of multivariate time series with small dimensions. Moreover, depending on the specific characteristics of the sampled graph and the number of nodes, alternative decaying functions, such as $\frac{1}{1+\sqrt{x}}$, $\frac{1}{1+x^{2}}$, exp(−*x*), need to be tested as they may yield improved results. These functions exhibit varying decay rates for the mixture weights, offering flexibility and adaptability in different scenarios.

Let the *P*-dimensional observed vector $Y\left(t\right)={\left[Y_{1}\left(t\right),\ldots ,Y_{P}\left(t\right)\right]}^{T}$. Then we have the following Cramer representation:


(6) $$Y(t)\;=\int_{-1/2}^{1/2}\exp(i2\pi \omega t)dX(\omega ),$$

where the *X*(ω) is a *P*-variate random process whose mean is zero with orthogonal increments having the following covariance:


(7) $$\begin{array}{ll} Cov(dX(\omega ),dX(\lambda )) & =\{\begin{array}{l} f(\omega )d\omega d\lambda \text{ if }\omega =\lambda +2\pi k,\;k\text{ an integer},\\ 0,\text{ otherwise.} \end{array} \end{array}$$

and *f*(ω) is the spectral density matrix. If we define the filtered components at band Ω to be:


(8) $$\begin{array}{ll} Y_{1,\Omega }\left(t\right) & =\underset{\ell }{\sum }{\text{Ψ}}_{\ell }Y_{1}\left(t-\ell \right), \end{array}$$

$$\begin{array}{l} \vdots \end{array}$$

(9) $$\begin{array}{ll} Y_{P,\Omega }\left(t\right) & =\underset{\ell }{\sum }{\text{Ψ}}_{\ell }Y_{P}\left(t-\ell \right), \end{array}$$

where the filter Ψ is the band pass filter centered around frequency band Ω. In Ombao and Van Bellegem (2008), coherence between *Y*_1_(.) and *Y*_2_(.) at frequency band Ω is derived to be the squared correlation between the phase-adjusted *Y*_1, Ω_(*t*) and *Y*_2, Ω_(*t*). Coherence will then be used (via a decreasing transformation) to define frequency-specific distance between time series components *Y*_*p*_(.) and *Y*_*q*_(.).

Consider the observed data {*Y*(*t*), *t* = 1, …, *T*}. The spectral matrix *f*(ω) can be estimated parametrically (e.g., by fitting a VARMA model), non-parametrically (by smoothing the periodogram) or semi-parametrically. In our case we will be using the smoothed periodogram approach. The Fourier *P*-dimensional coefficient at frequency ω_*k*_ are defined as:


(10) $$\begin{array}{ll} d\left(\omega_{k}\right) & =\frac{1}{\sqrt{T}}\overset{T}{\underset{t=1}{\sum }}Y\left(t\right)\exp\left(-i\omega_{k}t\right), \end{array}$$

then the Fourier periodogram is defined to be:


(11) $$\begin{array}{ll} I\left(\omega_{k}\right) & =d\left(\omega_{k}\right){d\left(\omega_{k}\right)}^{*}, \end{array}$$

where the ^*^ operator in represents the conjugate transpose. Consequently, *I*(ω_*k*_) is a *P*×*P* matrix. It can be shown that the periodogram *I*(ω_*k*_) is asymptotically unbiased. However, it is not a consistent estimator of the spectral matrix as the asymptotic variance does not decrease to zero even when we get more and more observations (i.e., *T* → ∞). Hence, we construct a mean-squared consistent estimator to be:


(12) $$\begin{array}{ll} \hat{f}\left(\omega_{k}\right) & =\underset{\omega }{\sum }k_{h}\left(\omega -\omega_{k}\right)I\left(\omega \right) \end{array}$$

where *k*_*h*_(ω−ω_*k*_) is a non-negative smoothing kernel centered around ω_*k*_ and *h* is the bandwidth parameter. In order to derive our distance function, first we define coherence as follows:


(13) $$\begin{array}{ll} C\;{(Y}_{p}\left(.\right),Y_{q}\left(.\right),\omega )\; & =\frac{|{\hat{f}}_{p,q}\left(\omega \right){|}^{2}}{{\hat{f}}_{p,p}\left(\omega \right){\hat{f}}_{q,q}\left(\omega \right)}\in \left[0,1\right], \end{array}$$

then we define the dependence-based frequency-specific distance function to be a decreasing function, for example, G(x)=1-x:


(14) $$\begin{array}{ll} D\;{(Y}_{p}\left(.\right),Y_{q}\left(.\right),\omega )\; & =G\;(C\left(Y_{p}\left(.\right),Y_{q}\left(.\right),\omega \right)\;). \end{array}$$

In the following, using the ideas explained previously we start by generating multivariate time series data with specified dependence patterns. We explain how dependence information contained in the graph *G* can be encoded in the homology structure of connectivity network, using a first example with one main cycle then a second example with two main cycles in the dependence network.

### 2.3 Multivariate time series with cyclic patterns

Our aim is to simulate multivariate time series data with predefined patterns in the dependence network. These simulations serve multiple purposes: (1) conducting a statistical test by obtaining the distribution of a predefined test statistic under the null hypothesis through simulations, and (2) evaluating the performance of TDA methods, including mean-squared error of the estimator and the test's power for group comparisons as sample size increases or the discrepancy between group parameters widens.

The topology of the brain network is known to be organized according to principles that maximize the flow of information and minimize the energy cost for maintaining the entire network (Sporns, 2013; Pessoa, 2014; Hilgetag and Goulas, 2015; Muldoon et al., 2016). However, neurological or mental diseases may affect that organization by degrading the structural and functional connectivity of the brain (e.g., Alzheimer's disease, ADHD etc.) (Bassett and Bullmore, 2009; Henry et al., 2020; Fathian et al., 2022). Therefore, it is important to model these alterations in functional connectivity using time series models that can capture dependencies beyond pairwise nodes in a brain network. Here, we will develop a procedure for simulating multivariate time series with a given number of cycles that may reflect complexity in the brain functional network.

#### 2.3.1 One main cycle pattern

In this first example we generate a multivariate time series with one cycle in the dependence structure. In this setting, we will impose the time series components that are relatively close to each other to be more strongly dependent than components that are farther apart.

**Figure 4:**
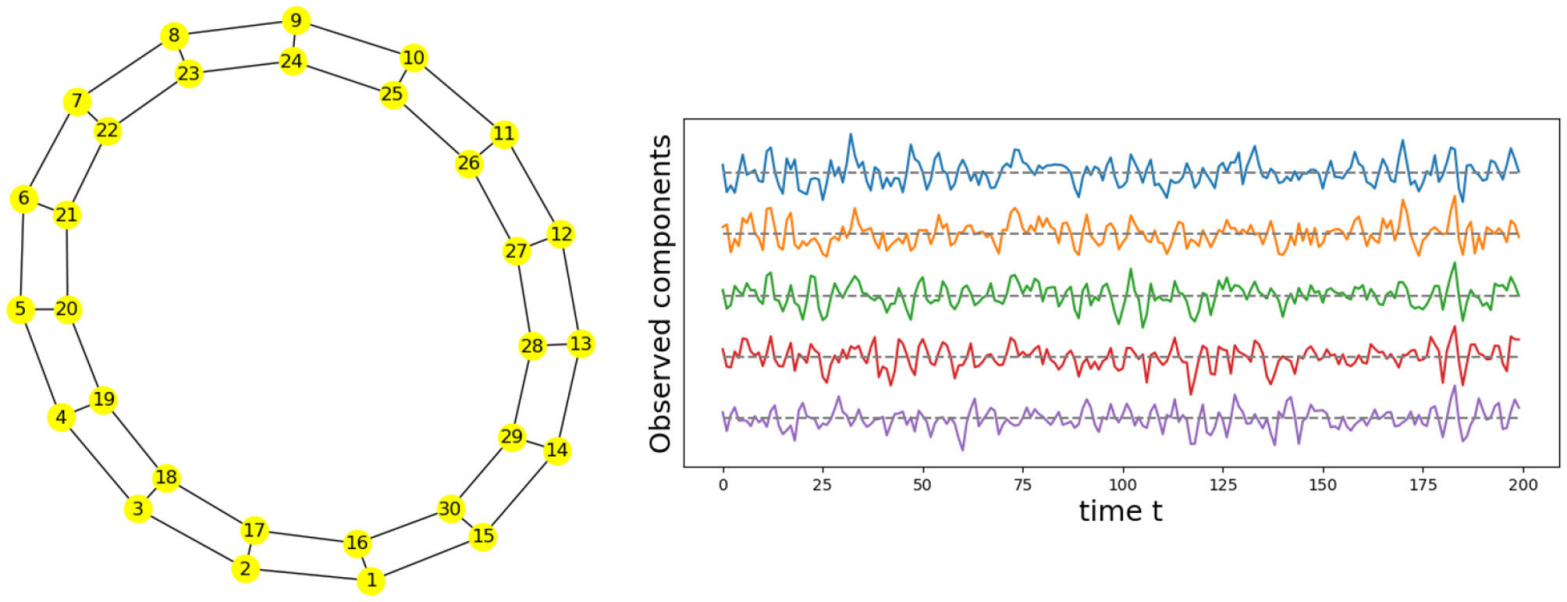
Cyclic dependence graph - Circular Ladder model **(Left)**. Generated time series with *P* = 30. The following components are plotted from top to bottom: *Y*_1_(*t*), *Y*_2_(*t*), *Y*_3_(*t*), *Y*_4_(*t*), *Y*_5_(*t*) **(Right)**.

Given the previous network definition of the circular ladder model, as displayed in Figure 4, we write the expression for *K* = 2, using Equation 4 for the observed time series $Y(t)={\left[Y_{1}(t),\ldots ,Y_{30}(t)\right]}^{T}$ and latent process $Z(t)={\left[Z_{1}(t),\ldots ,Z_{30}(t)\right]}^{T}$ as follows:


(15) $$\begin{array}{ll} Y\left(t\right) & =WZ\left(t\right)+\epsilon \left(t\right), \end{array}$$

where *W*_*p, q*_ is the contribution of the latent process *Z*_*q*_(*t*) in the observed process *Y*_*p*_(*t*) and is equal to $\frac{1}{1+d_{G}\left(p,q\right)}$ if the distance *d*_*G*_(*p, q*) between nodes *p* and *q* is less than or equal to 2 and 0 otherwise. Thus, the weight matrix *W* has dimension 30 × 30; the latent process vector *Z*(*t*) 30 × 1; and ϵ(*t*) is a 30 × 1 noise vector. Using this circular ladder model we can generate and visualize the multivariate time series data as follows in Figure 4. Given the model described above, we calculate pair-wise correlations for the following pairs: *Y*_1_(*t*)−*Y*_2_(*t*), *Y*_5_(*t*)−*Y*_6_(*t*), and *Y*_1_(*t*)−*Y*_6_(*t*). This calculation results in $Corr\;{(Y}_{1}\left(t\right),Y_{2}\left(t)\right)\;=Corr\;{(Y}_{5}\left(t\right),Y_{6}\left(t)\right)\;$, which simplifies to $\frac{10/6}{76/36}\sim 0.76$. Furthermore, $Corr\;{(Y}_{1}\left(t\right),Y_{6}\left(t)\right)\;=0$. Therefore, the correlation-distance between pairs 1-2 and 5-6 is the same 1 − 0.76, this is due to symmetry in the graph in Figure 4 and correlation-distance between component 1 and 6 is 1 because they do not share any latent components. Therefore, the time series components 1 and 24 are farther apart (based on the dependence distance) than 1 and 2 or 5 and 6, which is exactly the desired property.

**Figure 5:**
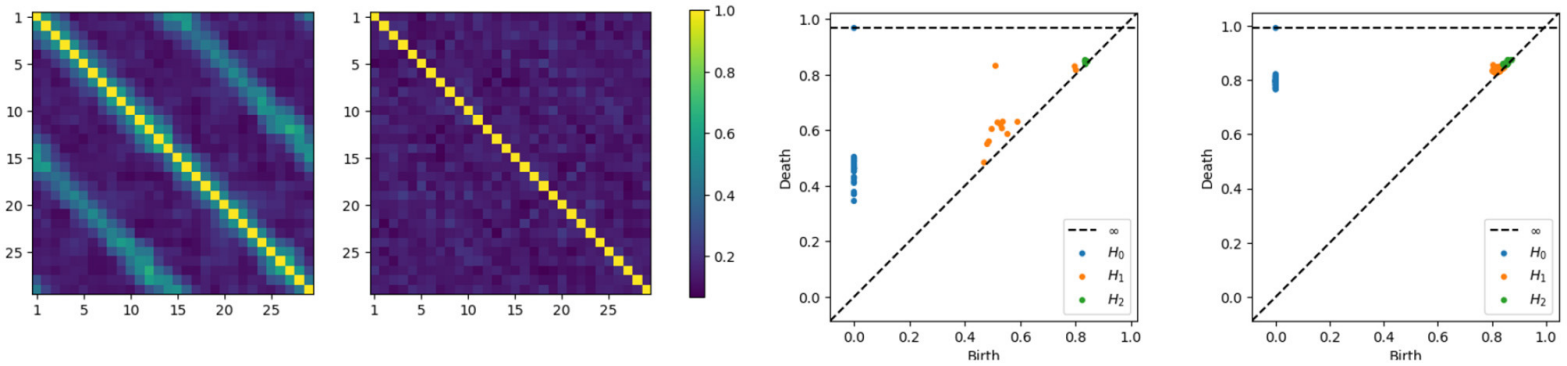
Coherence estimates for a one-cycle model. Leftmost: middle-frequency band. Second from the left: high-frequency band. Persistence diagrams for the same one-cycle model, with the middle-frequency band shown as the third from the left and the high-frequency band as the fourth from the left.

Having developed intuition behind the mechanism that generates the time series components, we now directly compute the coherence matrices for various frequency bands and analyze the topological patterns present in the resulting network. After computing, at each frequency band, the coherence matrix, we also consequently compute the distance matrix, we now build the Rips-Vietoris filtration and visualize the results in the persistence diagram as can be seen in Figure 5.

The orange point in the middle frequency persistence diagram, far from the diagonal, represents the main cycle in the dependence structure. Whereas the orange dots near the diagonal represent the secondary cycles that are present all around the network, see in Figure 5.

#### 2.3.2 Two main cycles pattern

**Figure 6:**
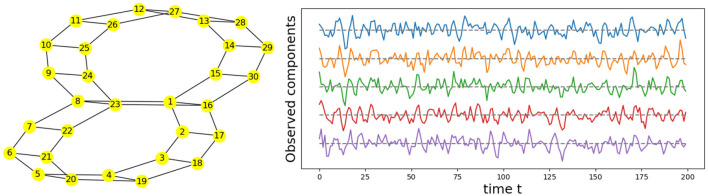
Cyclic dependence graph with two main cycles—double circular ladder model. Every node corresponds to a time series component **(Left)**. Generated time series with *P* = 30. The following components are plotted from top to bottom: *Y*_1_(*t*), *Y*_2_(*t*), *Y*_3_(*t*), *Y*_4_(*t*), *Y*_5_(*t*), derived from the double circular ladder model **(Right)**.

We now develop a model for generating multivariate time series with two cycles in the dependence structure. Similarly, from Equation 4, one can generate the multivariate time series with the double circular ladder model as defined in Figure 6. Using this mechanism we can generate and visualize the multivariate time series as follows (see Figure 6).

**Figure 7:**
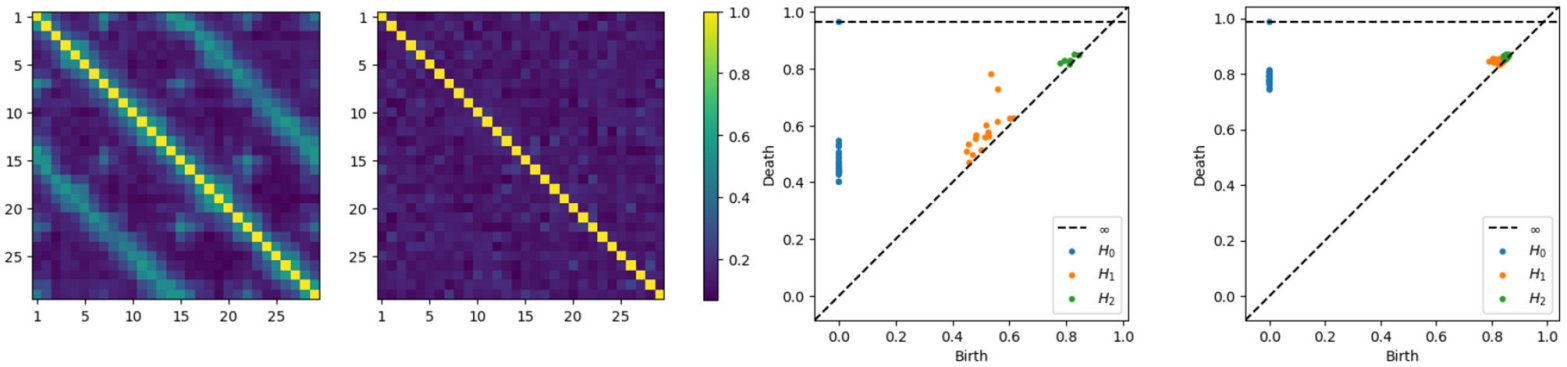
Coherence estimates for a two-cycle model. Leftmost: middle-frequency band. Second from the left: high-frequency band. Persistence diagrams for the same one-cycle model, with the middle-frequency band shown as the third from the left and the high-frequency band as the fourth from the left.

Without delving into the computational details of the coherence expression, it's important to note that in this new example, the coherence between any pair of channels within a subnetwork (one of the main cycles) will not reach zero, given the smaller diameter of the subcycles. Intuitively, components that are farther apart tend to exhibit weaker dependence. Therefore, having more connections shortens the path between nodes, resulting in increased dependence, which makes sense since more connections also mean more latent processes are being shared. On the other hand, components that are closer will exhibit stronger dependence as they share more latent processes. We can directly compute the coherence matrices for middle and high frequency bands and analyze the topological patterns present in the resulting network. After estimating the coherence matrices and therefore the distance matrices, the next step is to apply the tools of TDA, i.e., building the Rips-Vietoris filtration and visualize the results in the persistence diagram, as can be seen in Figure 7.

The x-axis represents the birth time (denoted *d*_*i*_), while the y-axis represents the death time (denoted *b*_*i*_), all the point representing valid features in the diagram have to lay above the diagonal line since the death time is larger than the birth time, i.e., *d*_*i*_>*b*_*i*_. The orange points far from the diagonal represent the two main cycles in the dependence structure. Whereas the orange dots near the diagonal represent the secondary cycles that are present all around the network, as can be seen in Figure 6.

**Figure 8:**
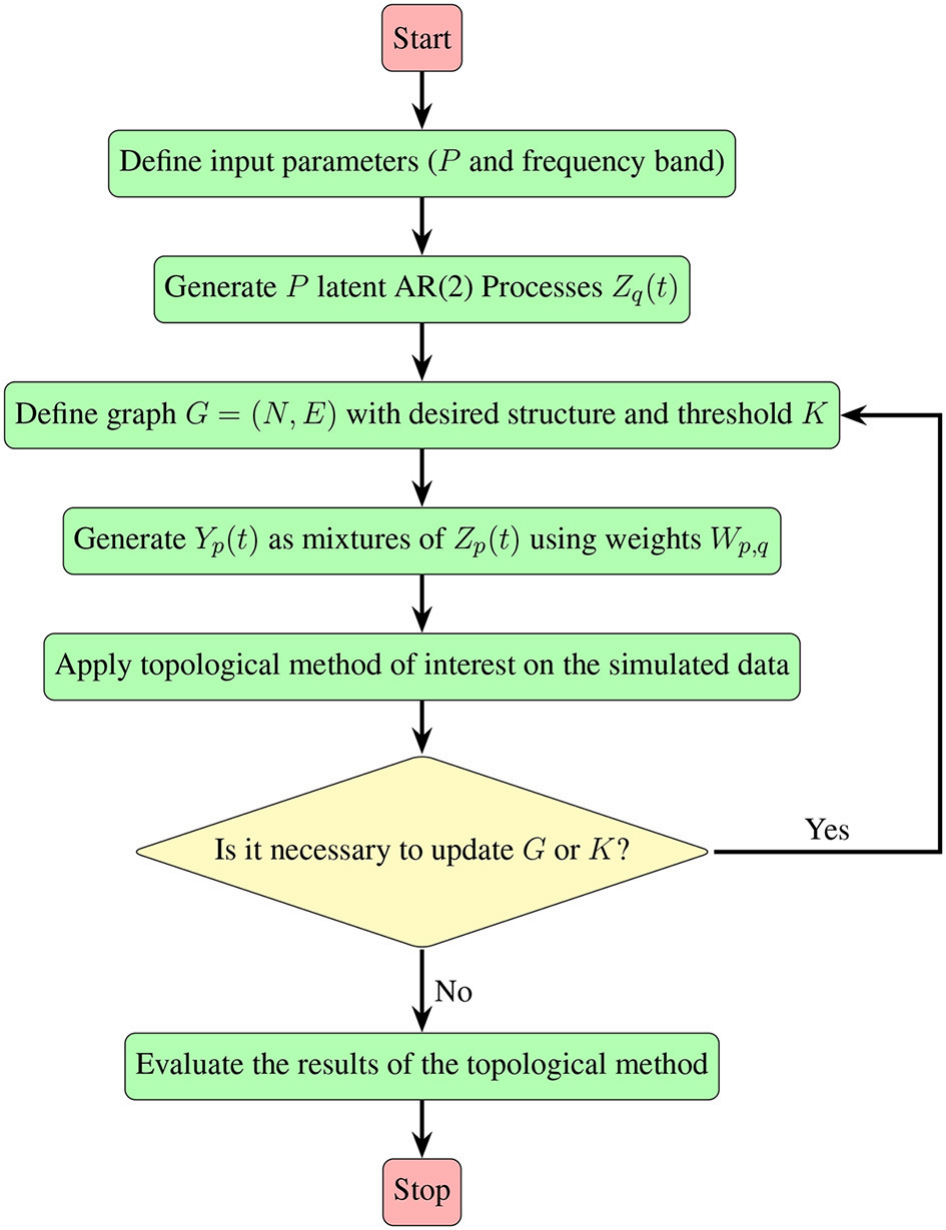
A visual guide demonstrating the simulation steps for multivariate time series, detailed in Section 2.2, along with the subsequent evaluation of the topological method of interest.

Irrespective of the specific pattern of interest, the approach detailed in Section 2.2, which leverages mixtures of AR(2) processes, proves to be versatile. It allows for the generation of multivariate time series data exhibiting diverse patterns within the dependence network, as long as the pattern can be represented by a graph. The following flowchart (Figure 8) provides an insightful summary of the procedural steps involved, contributing to a more accessible and intuitive understanding of our approach.

## 3 Generating multivariate time series with general patterns in its dependence network

**Figure 9:**
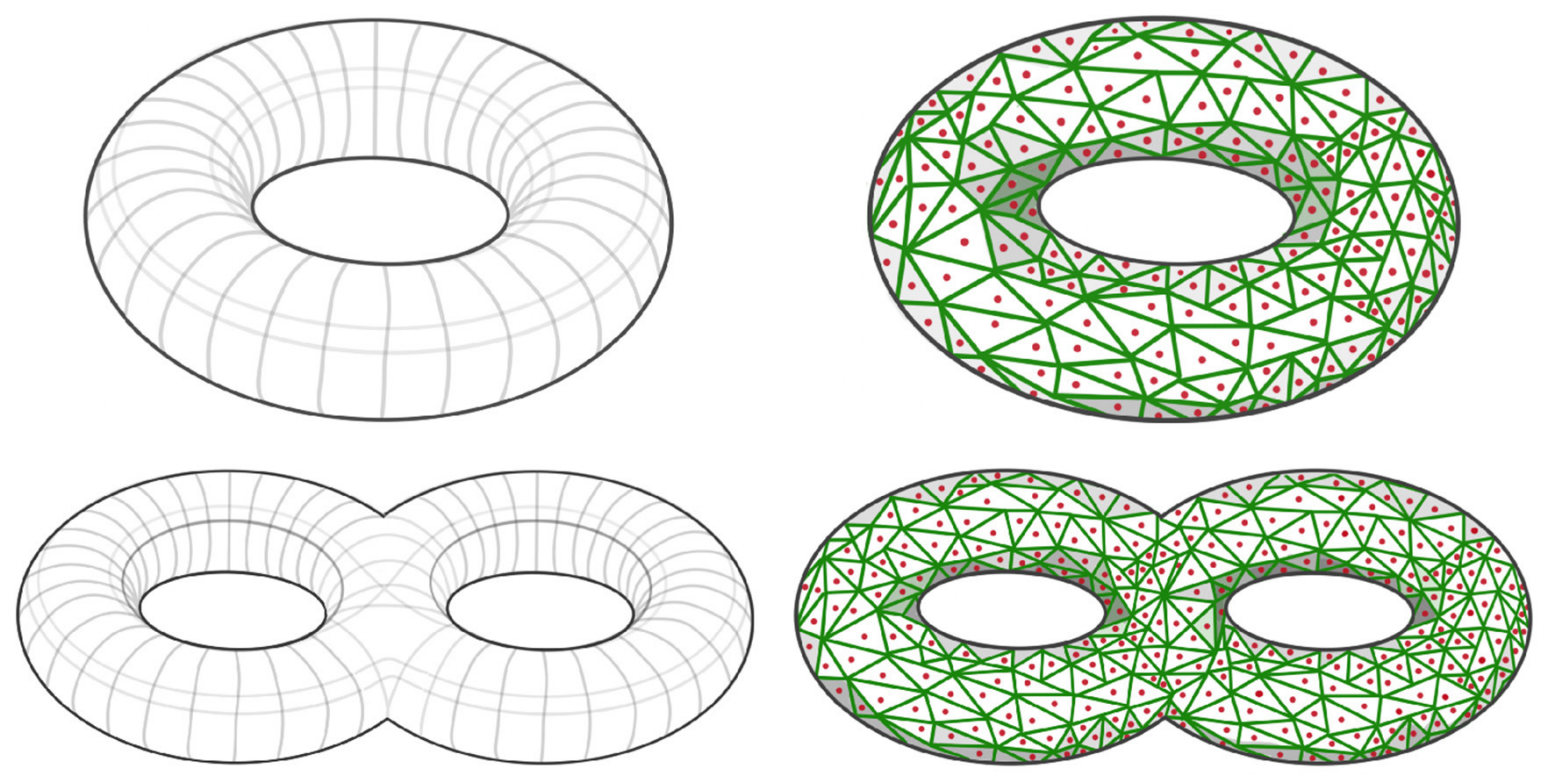
**(Top Left)** Simple torus dependence pattern; **(Top Right)** Sampled simple torus dependence pattern; **(Bottom Left)** Double torus dependence pattern; **(Bottom Right)** Sampled double torus dependence pattern.

Depending on the application of interest, the simulated patterns presented above may not be sufficient. However, the methodology is general and can be used to define many patterns in the dependence network of a multivariate time series. The goal in this section is to develop a novel robust procedure for simulating multivariate time series with complex dependence structures. Suppose that the interest is on a specific connectivity pattern, such as a torus or a double torus, see left hand side of Figure 9.

### 3.1 Defining the graph structure

After defining the shape of the manifold of reference M for the dependence structure, we need to define a graph $G_{M}=\left(N_{M},E_{M}\right)$ by sampling points *x*_*i*_ from such a topological structure (i.e., $x_{i}\in N_{M}\subset M$), as can be seen in red dots in right hand side of Figure 9. It is necessary to properly define this graph, because it is needed to compute the mixing weights in Equation 5, which are a (decaying) function of the graph distance between nodes. The right hand side of Figure 9 display a Voronoi tessellation (green lines) over the manifold. For every pair of nodes (region centers) that share a common border, we add an edge in the set of edges $E_{M}$ in the graph $G_{M}$. Once the graph is properly defined, as demonstrated in Figure 12, our next step involves generating a latent AR(2) process for each node within the graph. Subsequently, as shown in Figure 11, we create a new multivariate time series by employing the weighted approach described in Equation 4.

### 3.2 Sampling points from a manifold

In order to define the graph structure, it is necessary to have a mechanism to sample points uniformly from a manifold. Multiple sampling procedures have been proposed in the literature, for example (Diaconis et al., 2013; Soize and Ghanem, 2016; Baggenstoss, 2017; Prado and Ritto, 2021). It may be relatively straightforward to sample from simple manifolds, such as circles or spheres, because it is simple to parameterize the entire manifold, for instance using polar or spherical coordinates. However, generally 286 speaking, sampling from more intricate manifolds can be rather difficult. Uniformly sampling from manifolds extends beyond synthetic data generation and has broader implications in various domains. For instance, in the context of physical simulations, many systems have state spaces represented as manifolds. Achieving uniform sampling from these manifolds is critical for the effective study of system dynamics. Moreover, the relevance of uniform manifold sampling is apparent in biological and medical data analysis. Manifold representations are commonly used for complex data, including DNA structures, protein conformations, and brain functional connectivity. In these scenarios, non-uniform sampling can introduce biases into the analysis, impacting the quality and accuracy of results.

Our paper primarily focuses on two-dimensional surfaces, offering an novel approach based on quotient group representation for graph sampling with predefined patterns. This methodology contributes to solving the broader challenge of uniformly graph sampling from manifolds, which finds relevance in fields beyond synthetic data generation. The sampling problem (from a given manifold) is closely related to Bertrand's Paradox and the principle of indifference. Indeed, for such problem to display a unique solution one has to properly define the problem at hand and what is meant by sampling in a non-ambiguous way (Jaynes, 1973; Marinoff, 1994). For instance, considering the one dimensional circle embedded in ℝ^2^, every point $p_{1}\in M_{1}$ of the manifold $M_{1}$ can be represented by an angle θ:


(16) $$\begin{array}{ll} M_{1} & =\left\{\left(x,y\right)|x^{2}+y^{2}=r^{2}\right\}, \end{array}$$

(17) $$p_{1}\;=(r\cos(\theta ),r\sin(\theta )),\;\theta \in [0,2\pi ].$$

Similarly, considering the two dimensional sphere embedded in ℝ^3^, every point $p_{2}\in M_{2}$ can be represented by a pair of coordinates:


(18) $$\begin{array}{ll} M_{2} & =\left\{\left(x,y,z\right)|x^{2}+y^{2}+z^{2}=r^{2}\right\}, \end{array}$$

(19) $$\begin{array}{l} p_{2}=(r\sin(\theta )\cos(\phi ),r\sin(\theta )\sin(\phi ),r\cos(\theta )),\phi \in [0,2\pi ],\\ \;\theta \in [-\pi /2,\pi /2]. \end{array}$$

For instance, the parameterizations in Equation 17 correctly characterizes the circle. Hence, it is possible to sample points *p*_*i*_ from the manifold $M_{1}$ using the following procedure:


(20) $$\begin{array}{ll} \theta_{1} & \sim U\left(0,2\pi \right), \end{array}$$

(21) $$\begin{array}{ll} p_{1} & =\;(r\cos\left(\theta_{1}\right),r\sin\left(\theta_{1}\right)\;). \end{array}$$

Similarly, the parameterization in Equation 19 correctly characterizes the two dimensional sphere of radius *r*. Hence, to sample points *p*_*i*_ from $M_{2}$ we can use the following procedure:


(22) $$\begin{array}{ll} \theta_{2} & \sim U\left(0,2\pi \right), \end{array}$$

(23) $$\begin{array}{ll} \phi_{2} & \sim U\left(-\pi /2,\pi /2\right), \end{array}$$

(24) $$\begin{array}{ll} p_{2} & =\;(r\sin\left(\theta_{2}\right)\cos\left(\phi_{2}\right),r\sin\left(\theta_{2}\right)\sin\left(\phi_{2}\right),r\cos\left(\theta_{2}\right)\;). \end{array}$$

Both examples presented above rely on parameterized immersions. When the chosen parameterization f:S→M is not volume-preserving, the resulting sample will not be uniform. Indeed, this approach will lead to compressed regions being oversampled. Moreover, expanded regions can be undersampled, i.e., based on uniform sampling in the parameter space *S* the sampled points in M are denser in regions where the parameterization *f* has higher curvature (Diaconis et al., 2013). For example, in the first example the sample is uniform, however, in the second example the sample will not be uniform as there are compressed regions around the poles and expanded regions farther away from the poles. To remedy this issue, one potential approach is to generate a large sample using the previous approach then discarding some of the samples to correct for the compressed and expanded regions (see Diaconis et al., 2013). The rejection rate is chosen as a function of the determinant of the Jacobian of the parameterization f:S→M. Other interesting approaches have been proposed in the literature, such as Soize and Ghanem (2016) and Prado and Ritto (2021). However, these approaches provides tools for sampling only for simple manifolds.

When the manifold of interest is not very simple, such as a double torus in Figure 9 or even more complicated surfaces, it can be quite challenging to generate a sample using the above mentioned approach, since in some cases there may not be a global parameterization. Indeed, for smooth manifolds the parameterization is only guaranteed locally, to parameterize the entire manifold it is necessary to look at what is known as an Atlas representation of the manifold, refer to Tu (2008), for more details regarding the parameterization of manifolds.

**Figure 10:**
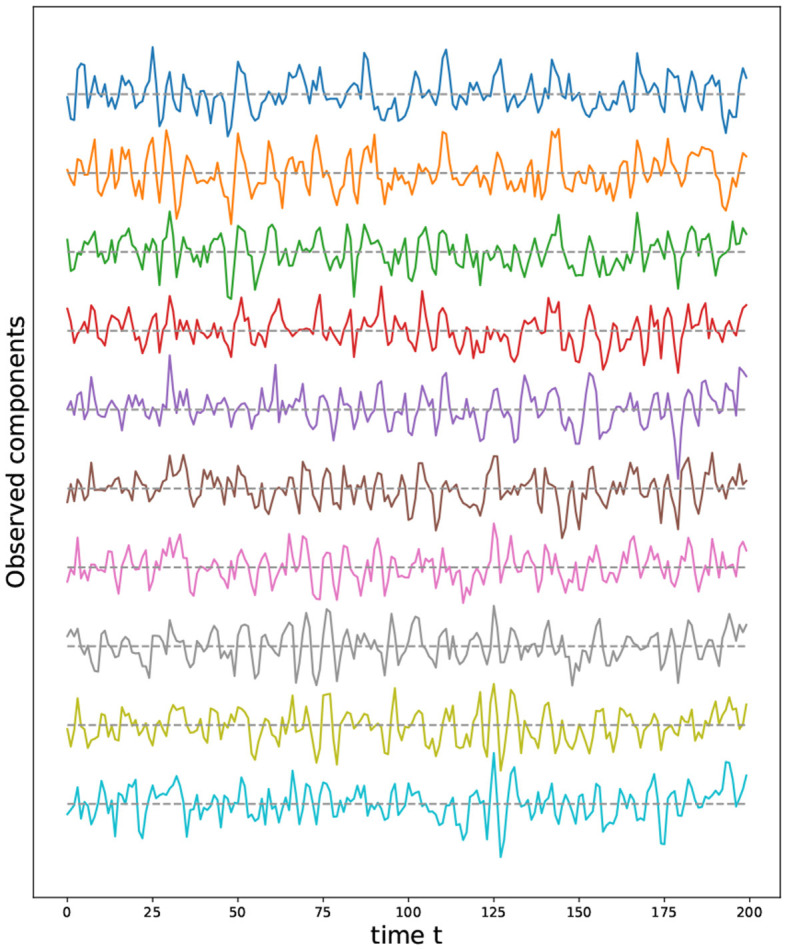
**(Left)** Polygonal quotient group representation for a cylinder, sphere, torus and a double torus. **(Right)** Corresponding 3D visualization.

For these reasons, we propose the following method to sample from a certain set of two-dimensional manifolds (surfaces), such as the sphere, torus, and double torus, which is based on the representation of these manifolds using quotient space of polygons (see Figure 10).

The advantage behind this proposed representation lies in the simplicity with which we can sample from the corresponding manifold. Indeed, given a polygonal representation, one can sample uniformly from the flat polygons, then identify the nodes present on equivalent edges. In Figure 12, we illustrate the process of constructing the graph representing a Torus manifold from the initial sample, taken from a rectangle, to the graph after node identification.

**Figure 11:**
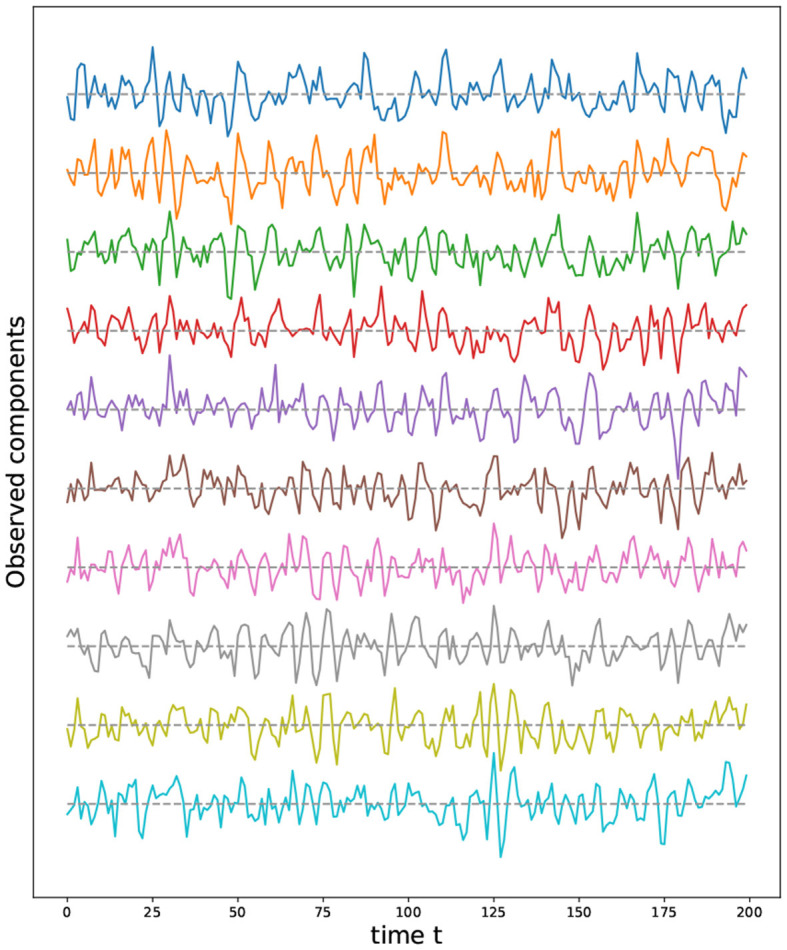
First ten time series components from a multivariate time series model generated using a torus structure with an initial grid of 9 by 17 nodes (i.e., *P* = 153).

**Figure 12:**
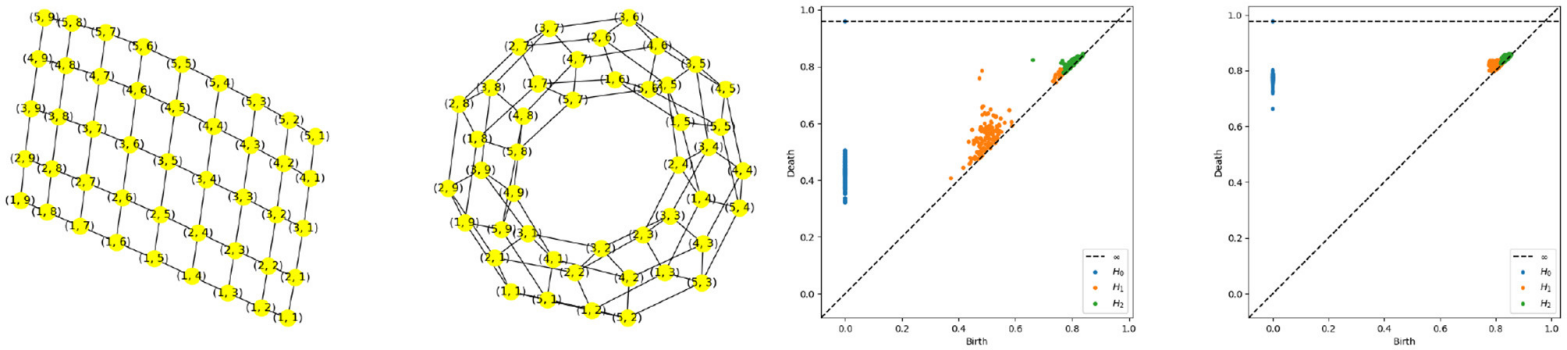
Torus graph pattern based on polygonal quotient group representation. Left most: Regular sample from a rectangle. Second from the left: Torus graph after node identification. Persistence diagram based on a multivariate time series generated using a torus structure with an initial grid of 9 by 17 nodes,i.e., *P* = 153, and *K* = 3, with the middle-frequency band shown as the third from the left and the high-frequency band as the fourth from the left.

Using the same approach as described in Equation 4, we generate the torus multivariate time series, as seen in Figure 11. After estimating the coherence matrix for this multivariate time series at middle and high frequency bands we compute the persistence diagrams and hence produce the following results, as displayed in Figure 12. This figure clearly shows the topological features of the targeted torus structure. Indeed, the two off-diagonal orange dots represent the two one dimensional wholes in a torus (circles surrounding each of the wholes) and one off-diagonal green dot representing the two-dimensional whole (or cavity inside of the torus), indeed it is known that the first three Betti numbers of a torus are β_0_ = 1, β_1_ = 2, β_2_ = 1.

The number of points to sample from the manifold depends on the target topological feature that is being investigated. Generally speaking, in order to detect features in the persistence diagram, the diameter of every subgraph surrounding the topological feature of interest needs to be at least of the same magnitude or larger than twice the constant *K* in the mixing equation. This is an important point to keep in mind. In Section 2.3.1, to detect the main cycle we need the diameter (*P*/4) to be larger than 2*K*, if *K* = 2 then we need to chose *P*≥16, if *K* = 3 then we need to chose *P*≥24 etc. In Figure 2.3.2, to detect the main cycles we need the diameter of the smallest subgraph surrounding one of the main cycles (roughly *P*/8 if both cycles are of comparable size) to be larger than 2*K*, i.e., if *K* = 2 then we need to chose at least *P*~32. For this reason, we can detect only the main cycles and the secondary cycles appear like noise in the persistence diagrams (see Figures 5, 7).

### 3.3 A robustness study: navigating noise effects

**Figure 13:**
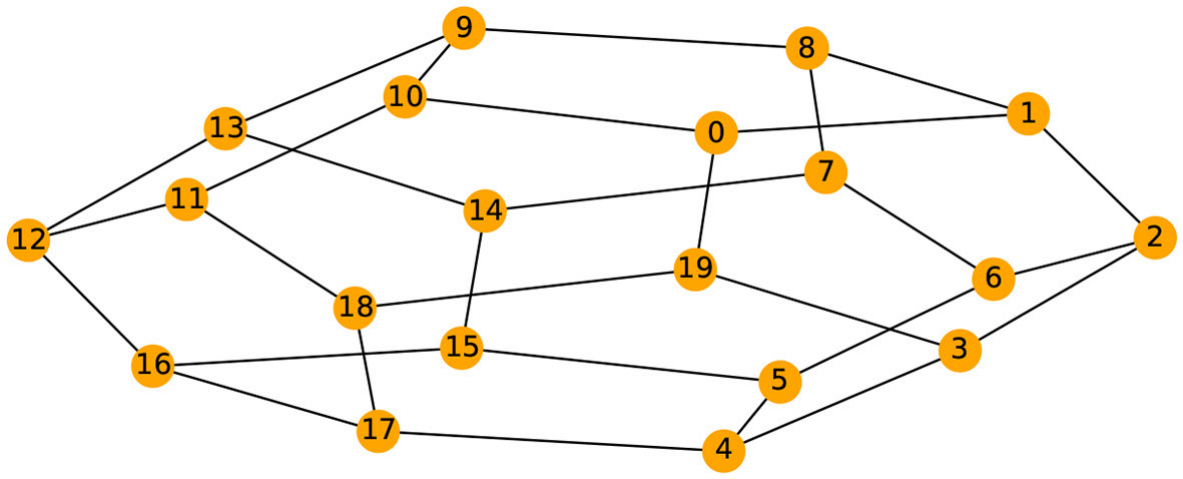
Example of a spherical dependence pattern known as a Dodecahedron.

**Figure 14:**
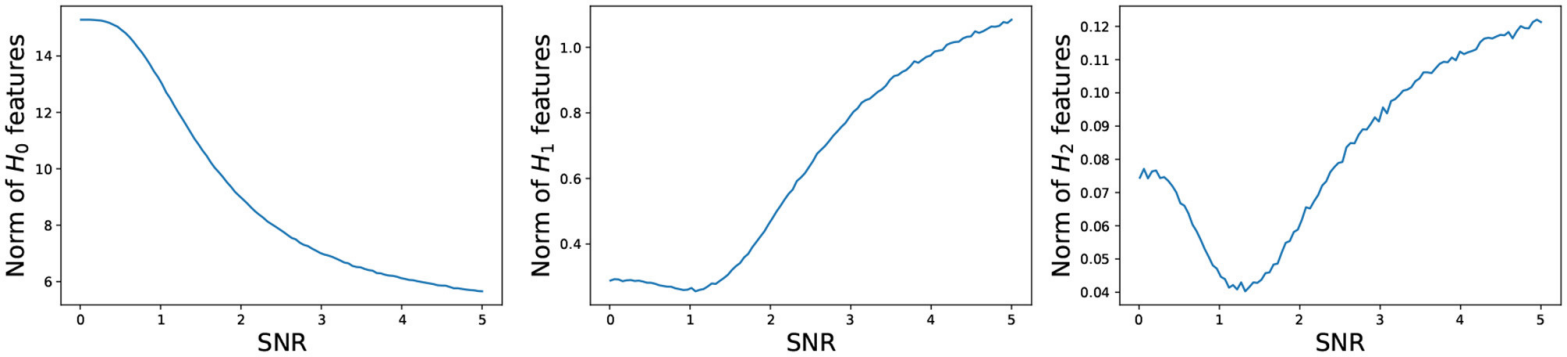
Total persistence as a function of the signal to noise ratio. **(Left)** 0-dimensional homology; **(Middle)** 1-dimensional homology; **(Right)** 2-dimensional homology. Total persistence in the *y*-axis and signal to noise ratio *SNR* in the *x*-axis. The plots are based on the average total persistence for 1,000 replicates.

Our aim here is to study the sensitivity of our approach to noise. The observed signal, denoted as *Y*(*t*), is composed of two components: the underlying signal or stochastic process, *S*(*t*), and additive noise, *N*(*t*). While *Y*(*t*) is what we directly observe, *S*(*t*) remains hidden from our measurements, characterized by a variance of $\sigma_{S}^{2}$. In contrast, *N*(*t*) is independent of *S*(*t*) and introduces noise with a variance of $\sigma_{N}^{2}$. To assess this sensitivity, we utilize the signal-to-noise ratio (*SNR*), defined as $SNR=\frac{\sigma_{S}^{2}}{\sigma_{N}^{2}}$, which quantifies the relative strength of the underlying signal to the additive noise. To assess the effect of the noise on the topological features of the dependence pattern in the underlying signal, we generate multivariate times series data from a structure that has two dimensional feature. i.e., a spherical structure (see Figure 13). Define the total persistence to be the norm of the persistence diagram' features as follows:


(25) $$\begin{array}{l} P_{k}=\underset{i\in PD_{k}}{\sum }\left(d_{i}^{k}-b_{i}^{k}\right) \end{array}$$

where $b_{i}^{k}$ and $d_{i}^{k}$ represent, respectively, the birth and death of the *i*-th *k*-dimensional topological feature in the persistence diagram. For every dimension *k*, the total persistence *P*_*k*_ is defined to be the sum of the persistence of all *k*-dimensional features in the persistence diagram. In what follows, we study the behavior of the total persistence *P*_*k*_ as a function of the signal to noise ratio (see Figure 14).

The persistence *P*_0_ of the 0-dimensional features decreases as the signal to noise ratio grows, which is to be expected because at low *SNR*, the time series components are mostly independent, resulting in large mutual distances and many unconnected components and at high *SNR*, the time series components are mostly dependent, resulting in smaller mutual distances, i.e., fewer connected components. On the other hand, the persistence *P*_*k*_ of the 1- and 2-dimensional features increases as the signal to noise ratio grows, which makes sense. At low *SNR*, the time series components are independent, and the connectivity pattern is not visible, but at high *SNR*, the time series components are mostly dependent according to the spherical structure, i.e., more 1- and 2-dimensional features.

## 4 Statistical inference in TDA—A simulation approach

There are many disorders that can alter the connectivity of the brain such as Alzheimer's disease, Parkinson's disease, ADHD. These conditions are known to alter the topology of the brain's connectivity structure by creating holes, cavities or other patterns in the connectivity network. We will develop a statistical inference method, via simulations of multivariate time series, for differentiating between two topological patterns that differ in their one dimensional homology structure. Based on the idea developed in Sections 2.3.1 and 2.3.2, we generate *N* = 50 samples from one model $M_{1}$ with one main cycle in its dependence pattern, and *N* = 50 samples from another model $M_{2}$ with two main cycles in its dependence pattern:


(26) $$\begin{array}{l} Y^{\left(1,i\right)}\left(t\right)=W^{\left(1\right)}Z^{1,i}\left(t\right)+\epsilon^{1,i}\left(t\right),i=1,\ldots ,N \end{array}$$

(27) $$\begin{array}{l} Y^{\left(2,i\right)}\left(t\right)=W^{\left(2\right)}Z^{1,i}\left(t\right)+\epsilon^{2,i}\left(t\right),i=1,\ldots ,N \end{array}$$

where *W*^(1)^ and *W*^(2)^ are respectively the mixing weights for model one and two as defined in Sections 2.3.1 and 2.3.2, *Z*^1, *i*^(*t*) and *Z*^2, *i*^(*t*) are the iid latent processes, ϵ^1, *i*^(*t*) and ϵ^2, *i*^(*t*) are the additive Gaussian noise.

After generating the time series for both groups, we compute the corresponding persistence diagrams then we compute a topological summary, total persistence as described in the previous section, i.e., $T_{i}^{1}$ and $T_{i}^{2}$ for *i* = 1, …, *N*. In order to compare the topologies of both groups we compute the group mean of these summaries for the one/two dimensional homology etc., and then assess the variability using a bootstrap approach:

1. Draw $T_{1}^{1*},\ldots ,T_{N}^{1*}$ and $T_{1}^{2*},\ldots ,T_{N}^{2*}$ from the empirical distribution based on $T_{1}^{1},\ldots ,T_{N}^{1}$ and $T_{1}^{2},\ldots ,T_{N}^{2}$
2. Compute the group mean ${\hat{T}}_{b}^{\left(1*\right)}=\frac{1}{N}\overset{N}{\underset{i=1}{\sum }}T_{i}^{1*}$ and ${\hat{T}}_{b}^{\left(2*\right)}=\frac{1}{N}\overset{N}{\underset{i=1}{\sum }}T_{i}^{2*}$ for the one and two homology groups.
3. Repeat B times the previous two steps.
4. Visualize the boxplot of the bootstrap samples.

**Figure 15:**
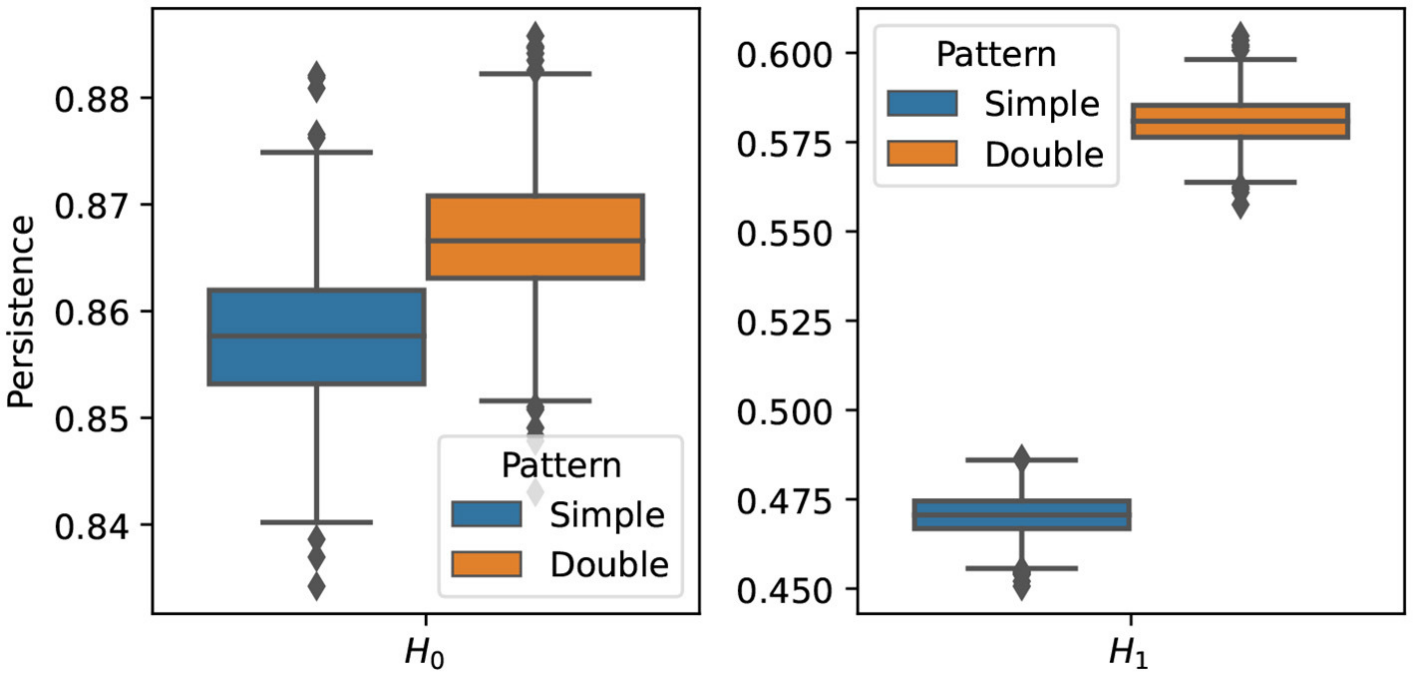
Boxplot of the one **(Left)** and two **(Right)** homology groups topological summaries for both group simple main cycle (blue) and double main cycle (orange) based on *B* = 1, 000 bootstrap samples.

The results of the procedure above is displayed in Figure 15. It can be seen that the two groups differ mainly in their cyclic structure (1-dimensional homology), high orange boxplot means more persistence of such features but not in their connected components structure (0-dimensional homology). In conclusion, based on the simulated data sets generated from models $M_{1}$ and $M_{2}$, it is possible to generate multivariate time series data with varying cyclic behavior in its dependence patterns.

## 5 Conclusion

This article presents an innovative approach for simulating multivariate time series data with predetermined cyclic dependency structures, which is crucial for evaluating the effectiveness of proposed Topological Data Analysis (TDA) techniques. To the best of our knowledge, our proposed method is the first to utilize mixtures of AR(2) processes to create frequency-specific dependency structures. Since our method is fairly general, it may be applied in a wide variety of situations. It can also be utilized to produce higher dimensional topological features. The proposed ideas were illustrated on examples with different cycle counts. A novel procedure based on the quotient group representation to create even more complex dependency patterns such as a torus is presented. To investigate the effect of the variance of the additive noise on the topological features, we conducted a thorough sensitivity analysis. Finally, we gave a demonstration of how our method can be applied to make simulation-based inference.

## Data availability statement

The data utilized in this study are simulated and generated for the purpose of experimentation. As such, they do not represent real-world observations. The simulation details and code used to generate the data are available upon request from the corresponding author.

## Author contributions

AE-Y: Conceptualization, Methodology, Software, Visualization, Writing – original draft, Formal analysis, Investigation, Validation. MC: Supervision, Writing – review & editing. HO: Funding acquisition, Resources, Supervision, Writing – review & editing.
